# Photodynamic Therapy for the Treatment of Bowen’s Disease: A Review on Efficacy, Non-Invasive Treatment Monitoring, Tolerability, and Cosmetic Outcome

**DOI:** 10.3390/biomedicines12040795

**Published:** 2024-04-03

**Authors:** Paolo Antonetti, Cristina Pellegrini, Chiara Caponio, Manfredo Bruni, Lorenzo Dragone, Mirco Mastrangelo, Maria Esposito, Maria Concetta Fargnoli

**Affiliations:** 1 Department of Biotechnological and Applied Clinical Sciences, University of L’Aquila, 67100 L’Aquila, Italy; paolo.antonetti@studenti.unich.it (P.A.); cristina.pellegrini@univaq.it (C.P.); manfredo.bruni@gmail.com (M.B.); mirco.mastrangelo@graduate.univaq.it (M.M.); maria.esposito3@univaq.it (M.E.); 2 Dermatology Unit, Ospedale San Salvatore, 67100 L’Aquila, Italy; chiara_caponio@hotmail.it

**Keywords:** photodynamic therapy, Bowen’s disease, squamous cell carcinoma in situ, MAL-PDT, ALA-PDT, non-melanoma skin cancer, immunocompromised patients

## Abstract

Bowen’s disease represents the in situ form of cutaneous squamous cell carcinoma; although it has an excellent prognosis, 3–5% of lesions progress to invasive cutaneous squamous cell carcinoma, with a higher risk in immunocompromised patients. Treatment is therefore always necessary, and conventional photodynamic therapy is a first-line option. The aim of this review is to provide an overview of the clinical response, recurrence rates, safety, and cosmetic outcome of photodynamic therapy in the treatment of Bowen’s disease, considering different protocols in terms of photosensitizers, light source, and combination treatments. Photodynamic therapy is a valuable option for tumors at sites where wound healing is poor/delayed, in the case of multiple and/or large tumors, and where surgery would be difficult or invasive. Dermoscopy and reflectance confocal microscopy can be used as valuable tools for monitoring the therapeutic response. The treatment is generally well tolerated, with mild side effects, and is associated with a good/excellent cosmetic outcome. Periodic follow-up after photodynamic therapy is essential because of the risk of recurrence and progression to cSCC. As the incidence of keratinocyte tumors increases, the therapeutic space for photodynamic therapy will further increase.

## 1. Introduction

Bowen’s disease (BD) is the intraepidermal (in situ) form of cutaneous squamous cell carcinoma (cSCC), first described by Bowen in 1912 [1,2]. An average annual incidence of 22.4 lesions/100,000 women and 27.8/100,000 men has been reported in the 5-year period from 1996 to 2000 in Canada [3], and of 142 lesions/100,000 Caucasian residents from 1983–1987 in Hawai [4]. The standardized incidence ratio for in situ carcinoma of the skin is 65 times higher in renal transplant recipients than in the general population [5]. Unlike cSCC, BD appears to have a slight prevalence in women [6]. Risk factors for BD are Fitzpatrick phototype I-II, age over 60 years, chronic UV exposure, and immunosuppression, similar to invasive cSCC [7,8]. Other recognized risk factors include arsenic exposure [9,10] and HPV infections [11].

**Figure 1:**
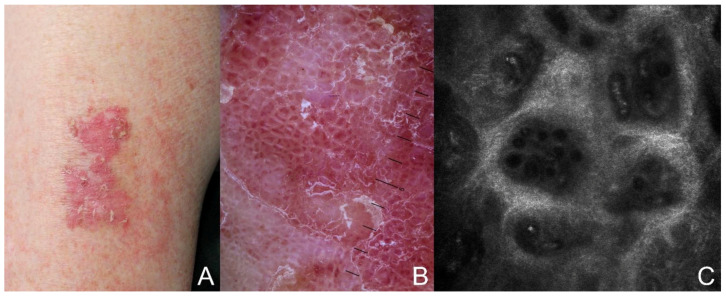
Clinical, dermoscopic, and confocal images of Bowen’s disease: (**A**) erythematous, scaly, well-demarcated plaque; (**B**) glomerular vessels and scaly surface on an erythematous base (10×); and (**C**) tightly coiled vessels, some with an S-shape, in the center of dermal papillae; hyper-reflective stroma (mosaic, 8 × 8 mm).

The classical clinical presentation of BD is an erythematous, scaly, slow-growing, well-demarcated patch or plaque, usually asymptomatic, although larger lesions may be associated with itching (Figure 1A). The overlying scales may be white or yellow and can be adherent or easily removed [12]. Less common variants include pigmented, subungual, periungual, palmar, perianal, and genital cSCC in situ, the latter referred to as Erythroplasia of Queyrat when it involves the penis. Regarding body sites, the most common anatomical sites for classic BD are the head and neck, followed by the limbs. The cheeks and lower limbs are more likely to be affected in women, while the bald scalp and ears are more often involved in men [13,14,15].

Dermoscopy can be used as quick and non-invasive diagnostic technique for BD. The presence of vascular structures, i.e., dotted or glomerular vessels, a scaly surface, and white structureless areas characterize BD on dermoscopy (Figure 1B) [16,17,18]. In the pigmented variant, additional dermoscopic findings include small brown globules regularly packed in a patchy distribution, reticular pigmentation and structureless gray to brown pigmentation. Additional non-invasive imaging tools such as reflectance confocal microscopy (RCM) (Figure 1C), conventional optical coherence tomography (OCT), and line-field confocal optical coherence tomography (LC-OCT) can help in the early diagnosis of keratinocyte skin tumors [19,20,21].

Histologically, as a carcinoma in situ, BD shows full-thickness epidermal involvement but does not extend beyond the basal membrane. It is characterized by atypical keratinocytes, sometimes multinucleated, associated with a disordered maturation of the epidermis, dyskeratotic cells, and mitosis at various levels. A loss of the granular layer is usually present, with overlying parakeratosis and sometimes hyperkeratosis. An involvement of the pilosebaceous apparatus is possible [3,22].

Keratinocyte carcinomas are well known to occur at a higher rate in immunocompromised patients, including organ transplant recipients (OTRs) as well as patients with other forms of immune suppression (chronic leukemias, infections, and autoimmune diseases). Much of the existing literature derives from studies on OTRs, which represent the majority of the immunocompromised population. The cumulative incidence is related to geographic latitude, skin type, and immunosuppressive therapies [23]. Indeed, the risk appears to be correlated with the level of immunosuppression in the transplant (heart > kidney > liver) [24]. A multicenter US retrospective cohort study including 10,649 adults receiving a primary organ transplant in 2003 or 2008 reported that 8% developed skin cancer, yielding an incidence ratio of 1437 per 100,000 person-years, and 94% of them were cSCC, yielding an incidence ratio of 1355 per 100,000 person-years [25]. Detailed data are limited on BD lesions. In a large Irish population-based study in renal transplant recipients, BD lesions represented 19% of all cancer types with a 65-fold increased standardized incidence ratio (SIR 64.6; 95% CI 53.7–75.5) [5].

The prognosis of BD is excellent, as it is usually a slow-growing lesion. However, the overall rate of progression to invasive cSCC is 3–5%, or even up to 10% for genital lesions [3,6,15,26], and is more common among the elderly and immunocompromised individuals [27,28]. Clinical signs suggestive of malignant transformation are ulceration, nodule formation, and bleeding [6].

Treatment options for BD are multiple, including surgical excision, cryotherapy, laser ablation, curettage with cautery, radiation therapy, topical 5% 5-fluorouracil (5-FU), imiquimod, and conventional photodynamic therapy (PDT). Factors to consider when choosing treatment include the number, site, size, and thickness of the lesions, as well as comorbidities, immune status, and patient’s preference [6,12,26]. Surgical excision is the first choice for the treatment of BD; however, non-invasive treatments are recognized as acceptable treatment options, with the opportunity to treat multiple lesions and the advantages of better cosmetic results and lower costs.

We performed a literature review on the application of PDT in the treatment of BD using the PubMed database, and the search terms were the following: photodynamic therapy, PDT, MAL-PDT, ALA-PDT, Bowen’s disease, and squamous cell carcinoma in situ. This review includes studies published through January 2024, describing clinical response, recurrence rates, cosmetic outcome, tolerability, and the adverse effects of PDT in the treatment of BD, considering different protocols in terms of photosensitizers, light source, and combination treatments.

## 2. PDT: Mechanism of Action and Treatment Protocol

PDT consists of the activation of a photosensitizing drug by visible light. The sensitizer, when irradiated, generates reactive oxygen species, such as singlet oxygen, the hydroxyl radical, the superoxide anion and hydrogen peroxide, which have a direct cytotoxic effect, and stimulate the release of immune mediators, resulting in additional pro-inflammatory effects [29,30]. In dermatologic indications, PDT uses precursors of the heme biosynthetic pathway, particularly 5-aminolaevulinic acid (5-ALA) or its ester, methyl aminolaevulinate (MAL), as photosensitizers, which are converted within target cells into protoporphyrin IX (PpIX) and activated through a light source.

Among the agents licensed for PDT in Europe, MAL (160 mg/g, Metvix^®^/Metvixia^®^, Galderma, Paris, France) is the only one authorized for use together with red light to treat cSCC in situ/BD. No formulation of ALA-PDT is licensed for this indication.

Current European guidelines recommend MAL-PDT for the treatment of BD with a strength of recommendation A and quality of evidence 1 [31]. PDT is particularly indicated for lesions at sites of poor healing, for large or multiple lesions, and in cases where surgery would be difficult or invasive such as facial, digital, nail bed, and penile lesions. The protocol involves two MAL-PDT sessions 7 days apart, repeated at 3 months, if necessary. It is advisable to prepare treatment sites by the gentle removal of overlying scales and crusts with saline-soaked gauze or a curette/scalpel. MAL is applied for 3 h under occlusion, and the treatment sites are then illuminated with an appropriate light source. The light source used is red light (630 nm), which has a greater ability to penetrate tissue than green or blue light, at a dose of 37 J/cm^2^ [31].

## 3. Efficacy of PDT in Monotherapy

The first large pan European study on MAL-PDT included 225 patients with histologically confirmed BD, randomized to MAL-PDT, cryotherapy, or topical 5% 5-FU for 4 weeks. After 3 months, lesion response rates were similar with all regimens (93% MAL-PDT, 86% cryotherapy, 83% 5-FU). At 12 months, the estimated sustained complete response rate with MAL-PDT was significantly higher than that with cryotherapy (80% vs. 67%, *p* = 0.047) and better than that with 5-FU (80% vs. 69%, *p* = 0.19). Maximum lesion diameter influenced the recurrence rate at 12 months after MAL-PDT, which was 10% in lesions up to 14 mm, 12% in lesions 15 to 29 mm, and 30% in lesions 30 mm or larger. However, response appeared to be independent of lesion location [32].

Overall, the clinical response rate for MAL-PDT in the treatment of BD varies from 88–100% after one or two cycles at 3 months, with 68–89% of lesions clear over follow-up periods of 17–50 months [32,33,34,35,36,37].

Calzavara et al. [33] investigated MAL-PDT for the treatment of BD and SCC, reporting an overall complete response rate of 87.8% at 3 months and 70.7% at 2 years in 41 biopsy-proven BD lesions [33]. In an observational, retrospective study, Truchuelo et al. [34] analyzed 51 BD tumors treated with MAL-PDT. The complete remission rate was 76.1%, and the recurrence rate was 14.3% after 1 year. A Swedish monocentric retrospective study on 423 BD lesions treated with MAL-PDT over a 13-year period found a complete response rate of 77.5% at the first follow-up visit (mean: 3.5 months) with a recurrence rate of 18.3% at the later follow-up visit (mean FU duration of 11.2 months, range 0.2–151 months) and an overall clearance rate of 63.4% [38]. The complete remission rates of small lesions (diameter < 20 mm) and large lesions (diameter > 20 mm) were 69.1% and 48.7%, respectively. A diameter greater than 20 mm was the main cause of treatment failure [38]. The other potential risk factors, i.e., gender, age, anatomic site, weeks between PDT sessions, and pain did not significantly correlate with the MAL-PDT efficacy rate.

Long-term MAL-PDT follow-up data have been reported in difficult-to-treat BD by Cavicchini and colleagues [35]. An analysis of 43 BD lesions showed a 100% complete response rate at 3 months and 89.4% at 50 months of follow-up. Jansen et al. [37] retrospectively studied 241 BD tumors treated with ALA or MAL-PDT (two sessions, one week apart) and found that the recurrence rate of BD tumors after 1 year and 5 years of PDT was 13.4% and 22.3%. In a large Spanish retrospective analysis of 537 BD lesions treated with MAL-PDT during the period 2006–2017, the 1-year and 5-year recurrence-free survival rates were 87% and 71%, respectively. Tumor size > 300 mm^2^ (≥21 mm in diameter), location on the upper extremities, and patient’s age <70 years were all associated with an increased risk of recurrence [39].

Clinical, histological, and immunohistochemical variables implicated in the response to MAL-PDT were analyzed in a retrospective study including 33 BD lesions [40]. A response to MAL-PDT was observed in 82% of the lesions after 3 months of follow-up, decreasing to 70% after 6 years of follow-up. The tumor size was significantly larger in nonresponders than in responders (25 ± 8.7 mm vs. 14.9 ± 7.6 mm). No histological variables were associated with the response to MAL-PDT. P53 immunostaining was positive in a higher proportion of responders as compared to non-responders, while cyclin D1 and EGFR immunostainings were more intense in non-responders. On a multivariate analysis, p53 was the only variable that significantly correlated with response to MAL-PDT, with a possible role in increasing PpIX levels and subsequent cell death [40].

Real-world experiences with ALA-PDT have been reported in small studies, describing a complete response rate of 80–100% in the short-term and a relapse rate of about 0–10% at 12 months [41,42,43]. This efficacy rate is consistent with either one or two treatments, with 10% or 20% ALA and using the single or two-fold illumination scheme. In a long-term follow-up study including 19 BD lesions treated with a single session of 20% ALA-PDT, 89.5% achieved complete clearance at 3 months, with 76.5% still clear at 2 years, but only 53.3% at 5 years [44].

A retrospective study including 68 BD lesions treated with 20% ALA solution and blue light, with variable incubation time and total number of PDT treatments, reported an initial complete response rate of 77.9% within 3 months after the completion of all PDT treatments, which was not associated with the number of PDT treatments. On multivariate analysis, a longer ALA incubation time, smaller tumor diameter (<2 cm), and location on the face were all associated with increased effectiveness of PDT [45].

Inconclusive data have been published regarding the comparison between ALA and MAL for PDT for BD. One study comparing the efficacy of ALA and MAL in 27 BD lesions found a complete response rate of 89% with ALA and 78% with MAL at approximately 6 months with no significant difference [46]. In a large study including 191 BD lesions, complete response was obtained in 84.7% of the lesions after ALA-PDT and in 55.1% after MAL-PDT (*p* < 0.001) at the 12-month follow-up [47].

**Figure 2:**
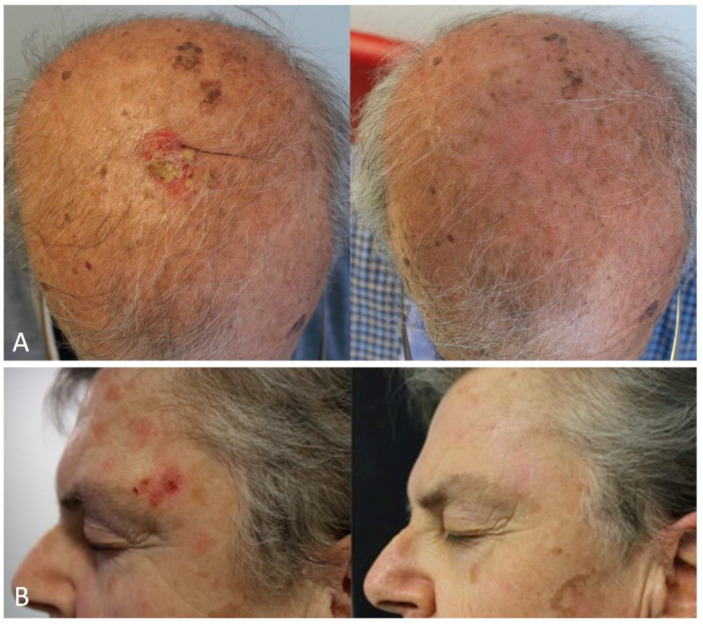
Treatment of Bowen’s disease with MAL-PDT. (**A**) BD lesion on the scalp in a 76-year-old OTR patient before and after two sessions of MAL-PDT, 1 week apart; (**B**) A 64-year-old female patient with a BD tumor on the temporal region before and after MAL-PDT treatment.

Table 1 summarizes relevant studies investigating PDT in the treatment of BD. None of the published studies separately analyzed the efficacy of PDT in treating BD lesions in sun-exposed and non-sun-exposed areas in terms of clinical response, recurrence rate, and cosmetic outcome. Figure 2 shows remission of BD after two sessions of MAL-PDT from our real life experience. 

A systematic review including nine studies assessed the different therapies for BD: PDT and 5-FU appeared effective, but due to limited evidence no clear conclusions on comparative efficacy were made. Surgical excision was not included because of the lack of comparative studies [36]. Later, Jansen et al. [37] retrospectively investigated the clinical efficacy of MAL or ALA-PDT, 5% 5-FU compared with surgical excision in 841 BD tumors. BD treated with 5-FU and PDT had a more than 2-fold increased 5-year probability of treatment failure compared with surgical excision, whereas there was no statistically significant difference between 5-FU and PDT. Of all treated BD, only eight tumors (seven post PDT and one post 5-FU) progressed into an invasive SCC, 3–42 months post-treatment. The same authors recently published a multicenter noninferiority trial comparing the effectiveness of 5% 5-FU cream twice daily for 4 weeks, 2 sessions of MAL-PDT with 1 week interval, and surgical excision in 250 patients with BD [53]. The proportion of patients with sustained clearance at 12 months was 97.4% after excision, 85.7% after 5-FU, and 82.1% after MAL-PDT. Based on the predefined noninferiority margin of 22%, 5-FU was noninferior to excision but associated with a better cosmetic outcome. For MAL-PDT, noninferiority to excision could not be concluded.

In a systematic review and meta-analysis including 12 randomized controlled trials published from 1996 to 2018, a higher lesion reduction rate after the first PDT treatment session was observed (OR = 2.86, 95%CI 1.89–4.33; *p* < 0.00001), with a significant difference versus both 5-FU (OR = 3.70; 95%CI: 2.07–6.62; *p* < 0.00001) and cryotherapy (OR = 2.24, 95%CI: 1.24–4.04; *p* = 0.008) [64]. However, no significant differences emerged in recurrence rates following treatment with PDT vs 5-FU (OR = 0.69; 95%CI 0.28–1.69) or cryotherapy (OR 0.53; 95%CI: 0.24–1.16). A more recently published meta-analysis including eight randomized controlled trials confirmed that PDT results in a significantly higher complete response rate (RR = 1.36, *p* = 0.04), reduced recurrences (RR = 0.53, *p* = 0.03), and better cosmetic outcomes (RR = 1.34, *p* = 0.0002) compared with other treatments, i.e., 5-FU and cryotherapy [65].

Evidence is very limited for daylight PDT (dlPDT). Two case reports of BD treated with dlPDT showed complete response in three BD lesions [51,66]. In a prospective study including 24 BD lesions treated with one cycle of 2 MAL-dlPDT sessions, complete clinical response was reported in 25% of the lesions, partial response in 57%, and no response in 16% of the lesions [52].

## 4. Combined Treatments

PDT has been combined with an ablative fractional resurfacing laser [55,61], CO_2_ laser [57], electrodessication [63], surgery [56], radiation [54], imiquimod [58], plum-blossom needle [60], and simple shaving [62] for BD treatment.

Combination therapy with laser-assisted techniques has been consistently demonstrated to effectively increase the penetration depth of the photosensitizer as well as increase PDT’s therapeutic effect. A small pilot randomized study supported the promising role of laser-assisted MAL-PDT in six BD lesions [59]. An efficacy of 80% was demonstrated with both continuous and fractional ablative CO_2_-assisted MAL-PDT after 12 months. PDT illumination was significantly less painful in the fractional-assisted MAL-PDT group. Ko et al. [55] compared the efficacy, recurrence rate, cosmetic outcome, and safety between a single treatment with Er:YAG ablative fractional laser-assisted PDT (AFL)-PDT and standard MAL-PDT (two treatment sessions with a 1-week interval) in 58 BD lesions. At 12 months, Er:YAG AFL-PDT was more effective than MAL-PDT (93.8% vs. 73.1%; *p* = 0.031) and the recurrence rate was significantly lower for Er:YAG AFL-PDT than MAL-PDT (6.7% vs. 31.6%; *p* = 0.022). No difference was found in terms of cosmetic outcome or safety [55]. A long-term follow-up study investigated the 5-year efficacy and recurrence rates of AFL-MAL-PDT and conventional MAL-PDT for the treatment of BD on the lower extremities in 84 lesions [61]. After 5 years, the overall clearance rate of AFL-MAL-PDT was significantly better than that of MAL-PDT (84.78% vs. 44.74%, *p* < 0.001). The recurrence rate was significantly lower for AFL-MAL-PDT than for MAL-PDT (9.3% vs. 41.38%, *p* = 0.003). Independent factors for treatment failure were a diameter larger than 20 mm and lesions previously treated.

Treatment with ALA-PDT combined with CO_2_ laser was compared to CO_2_ laser alone in a trial including 22 BD lesions. There was no difference in the complete remission rate (72.73% vs. 63.63%, *p* > 0.05); however, the recurrence rate at 6 months was significantly higher in the CO_2_ laser alone than in the ALA-PDT plus CO_2_ laser group (9.1% vs. 45.45%, *p* < 0.05) [57].

## 5. Non-Invasive Monitoring of Therapeutic Response

**Figure 3:**
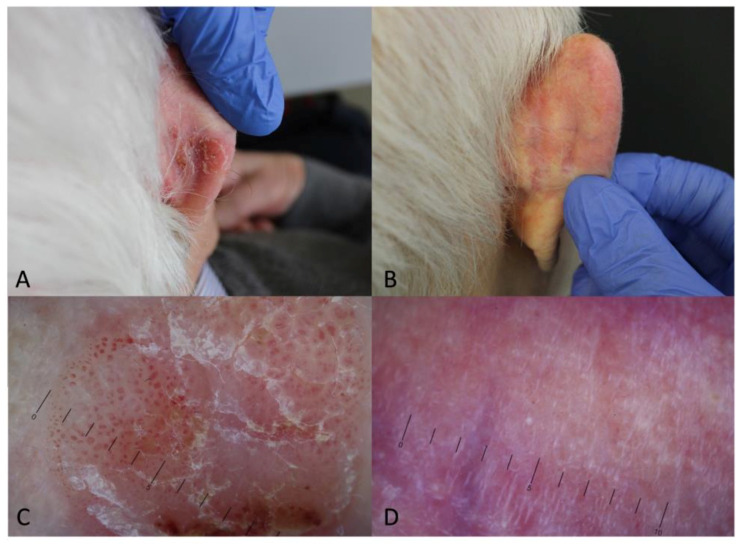
Dermoscopic monitoring (10×) of treatment response. Bowen’s disease in an 89-year-old patient on the retro-auricular area before (**A**–**C**) and after two sessions of MAL-PDT (**B**,**D**).

Dermoscopy can be used as a valuable follow-up tool in cases where non-surgical therapeutic options are chosen for the management of BD [17,67,68]. An illustrative example from our clinical experience is shown in Figure 3. Dermoscopic monitoring was performed 3 months after treatment in 23 patients with 29 histopathologically diagnosed BD lesions treated with MAL-PDT or imiquimod 5% cream [69]. Histopathological results showed that the cure rate for BD was 60% (3/5) for imiquimod cream and 50% (12/24) for MAL-PDT. After treatment, dermoscopic examination revealed the disappearance of pre-existing vascular structures in 16 lesions and residual vascular structures in 13 lesions. Histopathologic examination showed remnant intraepithelial neoplasias and increased vascularity in the dermis in lesions with persistent dermoscopic vascular structures. However, lesions without dermoscopic vascular structures showed normal epidermis and decreased vascularity in the dermis in all but one. During follow-up, one lesion showed a reappearance of vascular structures 9 months after treatment, which was confirmed to be a recurrence of BD after histopathology. These results supported the indications that emerged from a previous study by the same group [70].

RCM was useful to monitor for residual BD as well as to detect recurrence after PDT treatment in a case series including 10 patients with a total of 11 biopsies [71]. RCM imaging was found to help decrease unnecessary biopsies, especially in BD lesions that developed post-inflammatory erythema, which may make clinical and dermoscopic assessment difficult. In a case report, an in situ glans SCC was treated with two sessions of PDT using a copper bromide laser as a light source and the efficacy of the treatment was monitored with RCM [72]. After two sessions of PDT, RCM showed a normal mucosa, confirming the remission of the tumor.

## 6. Immunocompromised Patients

Immunocompromised BD patients appear to be significantly younger, more likely to have multiple tumors, and are at higher risk for recurrence and progression to invasive disease as compared to patients with normal immune function [27]. In addition, BD lesions occur multifocally and arise in body areas protected from UV light such as the trunk or the anogenital area.

Four BD lesions in transplant patients were treated with 1–2 sessions of ALA-PDT resulting in a complete response at 4 weeks; however, two patients experienced recurrence at 12 weeks [48]. One patient with two BD lesions underwent PDT with BF-200 ALA gel and red-light. The response (defined as an over 75% clearance of the lesion) was very good; however, incomplete resolution led to the recurrence of both lesions one year after treatment [73]. A randomized intrapatient comparative study found MAL-PDT more effective than 5% 5-FU in achieving a complete resolution of BD and AK lesions in eight OTR patients [49]. At 3-month follow up, the complete response rate for PDT was 89% (95% CI: 0.52–0.99), whilst for 5-FU it was 11% (95% CI: 0.003–0.48). At 6 months after treatment, the efficacy remained unchanged for both treatment groups. Unfortunately, the reported data do not allow discrimination between the response rates of BD and AK lesions.

Regarding the potential role of PDT in promoting the occurrence of SCC, a monocentric retrospective study investigated 105 patients with BD, including 25 (24%) immunocompromised patients, treated with MAL-PDT, who received a total of 151 different PDT fields. The efficacy of MAL-PDT was not significantly different between immunocompromised and non-immunocompromised patients. A total of 16 out of 105 patients developed SCC in PDT areas, after a median time of 6.0 months (IQR 2.7–11.8). The risk of the occurrence of at least one SCC in a PDT field was not significantly different between immunocompromised and non-immunocompromised patients [50].

Overall, limited evidence is available on the use of PDT for BD as well as on the comparison of PDT with other therapies in immunocompromised patients, making it difficult to draw conclusions; thus, treated patients should be closely monitored.

## 7. Tolerability and Cosmetic Outcome

Pain and burning during illumination, which peak in the first few minutes of treatment, are the main side effects of PDT. Expected skin phototoxicity effects are erythema, edema, vesiculation/pustulation, crusting, and erosion/ulceration. Long-term adverse effects such as pigmentary change, scarring or contact allergy, are uncommon. Systemic adverse events possibly related to the treatment have been very rarely reported [74].

Morton et al. [6] reported that most treatment-related events with MAL-PDT were considered as mild (60%) or moderate (34%), and only 6% were severe. By comparison, 12% of local events with cryotherapy were severe [6]. A higher severity of pain or burning during treatment, and of erythema after treatment, were observed in the MAL-PDT group compared to both excision and 5-FU (*p* < 0.001) [53]. When comparing MAL-PDT with ALA-PDT, no significant differences were identified in terms of high pain score (VAS, 8–10) (9% vs. 7%, respectively) and other frequent adverse events, such as erythema (41.9% vs. 43.6%), desquamation (37.5% vs. 32.7%), and superficial wounds (14% vs. 10.9%) [64].

In the study by Zaar et al. [38], the majority of BD lesions treated with MAL-PDT (195/250, 78.0%) healed with no long-term adverse events observed during follow-up. The most common adverse event was scarring, which was observed in 8.8% of the cases. Other local skin reactions were erythema (6%), hypopigmentation (2.4%), and hyperpigmentation (2.0%). Combinations of adverse events were seen in seven cases (2.8%).

The cosmetic outcome of MAL-PDT compares favorably with cryotherapy and 5% 5-FU in the treatment of BD lesions. At 3 months, MAL-PDT was superior to either cryotherapy or 5% 5-FU, with a good or excellent cosmetic outcome in 94% of patients vs. 66% for cryotherapy and 76% for 5% 5-FU, and was maintained at 12 months [6]. In addition, investigators and patients reported good/excellent outcomes significantly more often after MAL-PDT treatment than after excision (*p* < 0.001 and *p* = 0.006, respectively) [53].

## 8. Conclusions and Future Directions

PDT is a safe and effective, well-established treatment option for BD, especially in difficult locations, large or multiple lesions, and elderly patients. Lesion response appears to be significantly correlated with lesion size. Combination therapy with laser-assisted techniques has been shown to further improve PDT effectiveness. The published evidence on PDT both as monotherapy and combination therapy does not allow for adequate comparison because the protocols are different and the results regarding complete response and recurrence rate are variably reported. Noninvasive diagnostic techniques can help in the early diagnosis and treatment monitoring of BD. However, scientific evidence regarding treatment monitoring for BD is very limited, as it mainly focuses on actinic keratosis and basal cell carcinoma. Side effects, especially pain, are common, but generally mild, easily controlled, and self-limiting; patient information enables optimal management. The cosmetic outcome of MAL-PDT compares favorably with cryotherapy and 5% 5-FU with high levels of patient satisfaction. Patients with BD treated with PDT should be monitored after treatment because of the risk of incomplete response and recurrence, as well as progression to invasive cSCC, particularly for immunocompromised patients.

Considering the progressive aging of the general population, as well as the increase in immunosuppressed subjects, the incidence of both BD and cSCC is steadily increasing, constituting a growing public health problem. Future research on PDT for BD should focus on standardizing treatment protocols, improving the use of combination treatments, and encouraging studies of noninvasive methods in treatment monitoring, including those more recently introduced. Finally, for better patient selection, it would be desirable to promote large studies to identify additional predictors of clinical response and disease progression, beyond lesion size and immunosuppressive condition.

## Figures and Tables

**Table 1:** Relevant results from recent and/or larger studies on PDT for BD.

First Author, Year	No. of Patients	No. of Bowen’s Disease Lesions	Protocol	Clinical Response Rate (%)	Follow-up (Months)	Recurrence Rate	Cosmetic Outcome
Dragieva G, 2004 ^a^ [48]	4 (4)	4	20% ALA emulsion PDT, visible incoherent light, 75 J/cm^2^, 1 or 2 sessions	CR 100% at 1 month	12	50% at 12 months	Excellent in 100%
Morton C, 2006 [32]	96	111	MAL-PDT, red light 570–670 nm, 75 J/cm^2^, 2 sessions 1 week apart	CR 73% after two sessions and CR 93% after four sessions, at 3 months	12	15% at 12 months	Good or excellent in 97%
Perrett CM, 2007 ^a^ [49]	8 (8)	9	MAL-PDT, red light 633 ± 15 nm, 37 J/cm^2^, 2 sessions 1 week apart	CR 89% at 1 month	6	0 at 6 months	Excellent in 100%
De Haas ER, 2007 [42]	40	50	20% ALA ointment PDT, 1-fold illumination with diode 630 nm or LED light, 75 J/cm^2^, or 2-fold illumination with LED light, 20 and 80 J/cm^2^	CR 80%, single illumination CR 88%, 2-fold illumination at 12 months	24 (mean)	NA	Good in 92%
Calzavara-Pinton PG, 2008 [33]	NA	41	MAL-PDT, LED 635 nm, 37 J/cm^2^, 2 sessions 1 week apart	CR 87.8% at 3 months	24	17.1% at 24 months	Excellent in 62%
Souza CS, 2009 [44]	19	19	20% ALA emulsion PDT, 630 nm diode laser, 100 and 300 J/cm^2^, 1 session	CR 89.5% at 3 months	60	46.6% at 60 months	Good or excellent in 100%
Cavicchini S, 2011 [35]	30	43	MAL-PDT, red light 635 nm, 75 J/cm^2^, 2 sessions 1 week apart	CR 100% at 6 months	50 (mean)	11.6% at 12 months	Excellent in 100%
Truchuelo M, 2012 [34]	42	46	MAL-PDT, red light 630 nm, 38 J/cm^2^, 2 sessions 1 week apart	CR 76.1% PR 23.9%	16.6 (mean)	14.3% at 16.6 months (mean)	Excellent in 100%
Tarstedt M, 2016 [46]	NA	27	MAL-PDT or 20% ALA-PDT, red light 630 nm, 37 J/cm^2^, 1 or 2 sessions few weeks apart	CR 78% MAL-PDT CR 89% ALA-PDT, at 6 months	6	NA	NA
Ratour-Bigot C, 2016 ^a^ [50]	105 (25)	151	MAL-PDT, red light 570–670 nm, 37 J/cm^2^, 1 to 6 sessions	CR 52%, PR 26% At 3 months	14 (median)	NA	NA
Zaar, O 2017 [38]	335	423	ALA or MAL-PDT, red light 630 nm, 37–40 J/cm^2^, 2 sessions 1 week apart	CR 77.5% at 3.5 months (mean)	11.2 (mean)	18.3% at 11.2 months (mean)	Excellent in 78%
Jansen MHE, 2018 [37]	241	NA	ALA or MAL-PDT, red light 630 nm, 37–40 J/cm^2^, 2 sessions 1 week apart	NA	60	13.4% after 12 months; 22.3% after 60 months	NA
Gracia-Cazaña T, 2018 [40]	NA	33	MAL-PDT, red light 635 nm, 37 J/cm^2^, 2 sessions 1 week apart	CR 82% at 3 months	72	12% at 72 months	NA
Aguilar-Bernier M, 2019 [39]	NA	537	MAL-PDT, red light 630 nm, 37 J/cm^2^, 2 sessions 1 week apart	CR 88% at 12 months CR 71% at 60 months	33.2 (mean)	NA	NA
Alique-Garcìa S, 2019 [47]	171	191	MAL-PDT or 10% ALA gel, red light 635 nm, 37 J/cm^2^, 1 or 2 cycle of two sessions 12 weeks apart	CR 76.5% MAL-PDT CR 87.3% ALA-PDT, at 3 months	12	27.88% MAL-PDT 1.8% ALA-PDT at 12 months	NA
Safar R, 2019 [51]	1	1	Daylight MAL-PDT, 1 session	Complete remission	3	NA	Good
Kibbi N, 2020 [45]	58	68	20% ALA solution PDT, non-coherent blue light, 400–500 nm, 10 J/cm^2^, 1–4 sessions	CR 77.9% at 3 months	9.7 (median)	13.2% at 11.2 months (median)	NA
Martins CC, 2020 [52]	19	24	Daylight MAL-PDT, 2 sessions 1 week apart	CR 25%, PR 57% at 3 months	3	NA	NA
Cervantes JA, 2021 [43]	12	12	10% ALA gel PDT, 630 nm red light, 37 J/cm^2^, 1 or 2 sessions 10 days apart	CR 100% at 1 month	1	NA	Good or excellent in 75.3%
González-Guerra E, 2023 ^a^ [41]	1 (1)	2	BF-200 ALA gel PDT, red light 630 nm, 37 J/cm^2^, 3 sessions	PR 100% after treatment	12	100% at 12 months	NA
Ahmady S, 2024 [53]	78	78	MAL-PDT, red light 630 nm, 37 J/cm^2^, 2 sessions 1 week apart	CR 82.1% at 12 months	12	NA	NA
**COMBINED TREATMENTS**							
Nakano A, 2011 [54]	4	4	20% ALA solution PDT, excimer-pumped dye laser radiation,630 nm, 50 J/cm^2^ + Radiotherapy (3 Gy)	CR 100% at 3 months	14 (mean)	0 in 14 months (mean)	NA
Ko DY, 2013 [55]	21	58	MAL-PDT red light 632 nm, 37 J/cm^2^ + Er:YAG ablative fractional laser, one session or MAL-PDT red light 632 nm, 37 J/cm^2^, 2 sessions 1 week apart	CR 93.8% Er:YAG AFL-MAL-PDT CR 73.1% MAL-PDT at 3 months	12	6.7% Er:YAG AFL-MAL-PDT 31.6% MAL-PDT at 12 months	Excellent or good in 90.6% in the Er:YAG AFL-PDT group and 92.3% in the MAL-PDT group
Lu Y, 2014 [56]	13	13	Surgery + 10% ALA emulsion PDT, laser light 635 nm, 120 J/cm^2^, 3 sessions	CR 100% at 6 months	12	0 at 12 months	NA
Cai H, 2015 [57]	10	11	20% ALA emulsion PDT, red light 630 nm, 180 J/cm^2^ + CO_2_ laser 2–3 W, 1–3 sessions	CR 72.7%, PR 27.3% at 1 month	6	9.1% at 6 months	NA
Victoria-Martìnez AM, 2017 [58]	10	13	MAL-PDT or 10% ALA nano-emulsion PDT, red light 632 nm, 37 J/cm^2^, 3 sessions 1 week apart + Imiquimod 5% cream	CR 84.6%, PR 15.4% ^b^ at 3 months	18	18.1% at 18 months ^b^	Very good in 100%
Genouw, E 2018 [59]	6	6	MAL-PDT, red light 635 nm, 37 J/cm^2^ + CL (12 W) or FL (30 W) CO_2_ laser, 2 sessions 2 weeks apart	CR 80%, PR 20% at 12 months	12	NA	Good or excellent in 100%
Wu Y, 2018 [60]	24	38	PBN-ALA-PDT or ALA-PDT, 10% ALA cream, LED, 633 nm, 100–200 J/cm^2^	CR 77.8% PBN-ALA-PDT CR 40% ALA-PDT, at 1.5 months	12	0 PBN-ALA-PDT 11.7% ALA-PDT at 6 months	NA
Kim HJ, 2018 [61]	60	84	MAL-PDT red light 632 nm, 37 J/cm^2^ + Er:YAG ablative fractional laser, one session or MAL-PDT red light 632 nm, 37 J/cm^2^, 2 sessions 1 week apart	CR 93.48% Er:YAG AFL-MAL-PDT CR 76.3% MAL-PDT, at 3 months	60	9.3% Er:YAG AFL-MAL-PDT 41.38% MAL-PDT at 60 months	NA
Liu D, 2019 [62]	10	44	Simple shaving + 20% ALA cream PDT, 633 nm red light, 3 sessions 1 week apart	CR 100% at 3 months	12 (minimum)	0 at 12 months	Excellent in 100%
Liu X, 2023 [63]	11	12	Electrodesiccation + 20% ALA cream PDT, YAG LED-IB light, 633 nm ± 10 nm, 3 sessions	CR 100% at 12 months	17.5 (mean)	0 at 12 months	NA

*ALA*, aminolevulinic acid; *PDT*, photodynamic therapy; *MAL*, methyl aminolevulinate; *NA*, not available; *CR,* complete response, *PR*, partial response; *LED*, light emitting diode; *Er:YAG laser*, erbium-doped yttrium aluminium garnet laser; *CO_2_ laser*, carbon dioxide laser; *CL*, continuous ablative laser; *FL*, fractional ablative laser; *AFL*, ablative fractional laser; *PBN*, plum-blossom needling. ^a^ The study included immunosuppressed patients. The number of immunocompromised patients is reported in the brackets. ^b^ Patients with partial response or recurrence were treated with topical 5% imiquimod.

## References

[1] Bowen J.T. (1912). Precancerous dermatoses: A study of two cases of chronic atypical epithelial proliferation. J. Cutan. Dis..

[2] Callen J.P. (1988). Bowen’s disease and internal malignant disease. Arch. Dermatol..

[3] Arlette J.P., Trotter M.J. (2004). Squamous cell carcinoma in situ of the skin: History, presentation, biology and treatment. Australas. J. Dermatol..

[4] Reizner G.T., Chuang T.Y., Elpern D.J., Stone J.L., Farmer E.R. (1994). Bowen’s disease (squamous cell carcinoma in situ) in Kauai, Hawaii. A population-based incidence report. J. Am. Acad. Dermatol..

[5] Moloney F.J., Comber H., O’Lorcain P., O’Kelly P., Conlon P.J., Murphy G.M. (2006). A population-based study of skin cancer incidence and prevalence in renal transplant recipients. Br. J. Dermatol..

[6] Morton C.A., Birnie A.J., Eedy D.J. (2014). British Association of Dermatologists’ guidelines for the management of squamous cell carcinoma in situ (Bowen’s disease) 2014. Br. J. Dermatol..

[7] Kim J.Y.S., Kozlow J.H., Mittal B., Moyer J., Olenecki T., Rodgers P. (2018). Guidelines of care for the management of cutaneous squamous cell carcinoma. J. Am. Acad. Dermatol..

[8] Stratigos A.J., Garbe C., Dessinioti C., Lebbe C., Bataille V., Bastholt L., Dreno B., Fargnoli M.C., Forsea A.M., Frenard C. (2020). European interdisciplinary guideline on invasive squamous cell carcinoma of the skin: Part 1. epidemiology, diagnostics and prevention. Eur. J. Cancer.

[9] Miki Y., Kawatsu T., Matsuda K., Machino H., Kubo K. (1982). Cutaneous and pulmonary cancers associated with Bowen’s disease. J. Am. Acad. Dermatol..

[10] Watson K., Creamer D. (2004). Arsenic-induced keratoses and Bowen’s disease. Clin. Exp. Dermatol..

[11] Meyer T., Arndt R., Christophers E., Nindl I., Stockfleth E. (2001). Importance of human papillomaviruses for the development of skin cancer. Cancer Detect. Prev..

[12] Palaniappan V., Karthikeyan K. (2022). Bowen’s Disease. Indian Dermatol. Online J..

[13] Kossard K., Rosen R. (1992). Cutaneous Bowen’s disease. J. Am. Acad. Dermatol..

[14] Cox N.H. (1994). Body site distribution of Bowen’s disease. Br. J. Dermatol..

[15] Hansen J.P., Drake A.L., Walling H.W. (2008). Bowen’s Disease: A four-year retrospective review of epidemiology and treatment at a university center. Dermatol. Surg..

[16] Zalaudek I., Argenziano G., Leinweber B., Citarella L., Hofmann-Wellenhof R., Malvehy J., Puig S., Pizzichetta M.A., Thomas L., Soyer H.P. (2004). Dermoscopy of Bowen’s disease. Br. J. Dermatol..

[17] Lallas A., Argenziano G., Zendri E., Moscarella E., Longo C., Grenzi L., Pellacani G., Zalaudek I. (2013). Update on non-melanoma skin cancer and the value of dermoscopy in its diagnosis and treatment monitoring. Expert Rev. Anticancer. Ther..

[18] Ianoși S.L., Batani A., Ilie M.A., Tampa M., Georgescu S.R., Zurac S., Boda D., Ianosi N.G., Neagoe D., Calina D. (2019). Non-invasive imaging techniques for the in vivo diagnosis of Bowen’s disease: Three case reports. Oncol. Lett..

[19] Ulrich M., Kanitakis J., González S., Lange-Asschenfeldt S., Stockfleth E., Roewert-Huber J. (2012). Evaluation of Bowen disease by in vivo reflectance confocal microscopy. Br. J. Dermatol..

[20] Shahriari N., Grant-Kels J.M., Rabinovitz H.S., Oliviero M., Scope A. (2018). Reflectance Confocal Microscopy Criteria of Pigmented Squamous Cell Carcinoma In Situ. Am. J. Dermatopathol..

[21] Cinotti E., Bertello M., Cartocci A., Fiorani D., Tognetti L., Solmi V., Cappilli S., Peris K., Perrot J.L., Suppa M. (2023). Comparison of reflectance confocal microscopy and line-field optical coherence tomography for the identification of keratinocyte skin tumours. Ski. Res. Technol..

[22] Bhawan J. (2007). Squamous cell carcinoma in situ in skin: What does it mean?. J. Cutan. Pathol..

[23] Granata S., Tessari G., Stallone G., Zaza G. (2023). Skin cancer in solid organ transplant recipients: Still an open problem. Front. Med..

[24] Ulrich C., Arnold R., Frei U., Hetzer R., Neuhaus P., Stockfleth E. (2014). Skin changes following organ transplantation: An interdisciplinary challenge. Dtsch. Arztebl. Int..

[25] Garrett G.L., Blanc P.D., Boscardin J., Lloyd A.A., Ahmed R.L., Anthony T., Bibee K., Breithaupt A., Cannon J., Chen A. (2017). Incidence of and Risk Factors for Skin Cancer in Organ Transplant Recipients in the United States. JAMA Dermatol..

[26] Fania L., Didona D., Di Pietro F.R., Verkhovskaia S., Morese R., Paolino G., Donati M., Ricci F., Coco V., Ricci F. (2021). Cutaneous Squamous Cell Carcinoma: From Pathophysiology to Novel Therapeutic Approaches. Biomedicines.

[27] Drake A.L., Walling H.W. (2008). Variations in presentation of squamous cell carcinoma in situ (Bowen’s disease) in immunocompromised patients. J. Am. Acad. Dermatol..

[28] Eimpunth S., Goldenberg A., Hamman M.S., Oganesyan G., Lee R.A., Hunnangkul S., Song S.S., Greywal T., Jiang S.I.B. (2017). Squamous Cell Carcinoma In Situ Upstaged to Invasive Squamous Cell Carcinoma: A 5-Year, Single Institution Retrospective Review. Dermatol. Surg..

[29] Henderson B.W., Dougherty T.J. (1992). How does photodynamic therapy work?. Photochem Photobiol..

[30] Calzavara-Pinton P.G., Venturini M., Sala R. (2007). Photodynamic therapy: Update 2006. Part 1: Photochemistry and photobiology. J. Eur. Acad. Dermatol. Venereol..

[31] Morton C.A., Szeimies R.M., Basset-Seguin N., Calzavara-Pinton P., Gilaberte Y., Haedersdal M., Hofbauer G.F.L., Hunger R.E., Karrer S., Piaserico S. (2019). European Dermatology Forum guidelines on topical photodynamic therapy 2019 Part 1: Treatment delivery and established indications—Actinic keratoses, Bowen’s disease and basal cell carcinomas. J. Eur. Acad Dermatol. Venereol..

[32] Morton C., Horn M., Leman J., Tack B., Bedane C., Tjioe M., Ibbotson S., Khemis A., Wolf P. (2006). Comparison of topical methyl aminolevulinate photodynamic therapy with cryotherapy or Fluorouracil for treatment of squamous cell carcinoma in situ: Results of a multicenter randomized trial. Arch. Dermatol..

[33] Calzavara-Pinton P.G., Venturini M., Sala R., Capezzera R., Parrinello G., Specchia C., Zane C. (2008). Methylaminolaevulinate-based photodynamic therapy of Bowen’s disease and squamous cell carcinoma. Br. J. Dermatol..

[34] Truchuelo M., Fernandez-Guarino M., Fleta B., Alcántara J., Jaén P. (2012). Effectiveness of photodynamic therapy in Bowen’s disease: An observational and descriptive study in 51 lesions. J. Eur. Acad. Dermatol. Venereol..

[35] Cavicchini S., Serini S.M., Fiorani R., Girgenti V., Ghislanzoni M., Sala F. (2011). Long-term follow-up of methyl aminolevulinate (MAL)-PDT in difficult-to-treat cutaneous Bowen’s disease. Int. J. Dermatol..

[36] Bath-Hextall F.J., Matin R.N., Wilkinson D., Leonardi-Bee J. (2013). Interventions for cutaneous Bowen’s disease. Cochrane Database Syst. Rev..

[37] Jansen M.H., Appelen D., Nelemans P.J., Winnepenninckx V.J., Kelleners-Smeets N.W.J., Mosterd K. (2018). Bowen’s Disease: Long-term Results of Treatment with 5-Fluorouracil Cream, Photodynamic Therapy or Surgical Excision. Acta Derm. Venereol..

[38] Zaar O., Fougelberg J., Hermansson A., Gillstedt M., Wennberg-Larkö A.M., Paoli J. (2017). Effectiveness of photodynamic therapy in Bowen’s disease: A retrospective observational study in 423 lesions. J. Eur. Acad. Dermatol. Venereol..

[39] Aguilar-Bernier M., Rodríguez-Barón D., Rivas-Ruiz F., Segura-Palacios J.M., de Troya Martín M. (2019). Long-term efficacy of photodynamic therapy with methyl aminolevulinate in treating Bowen’s disease in clinical practice: A retrospective cohort study (2006–2017). Photodermatol. Photoimmunol. Photomed..

[40] Gracia-Cazaña T., Salazar N., Vera-Álvarez J., Aguilera J., López-Navarro N., Herrera-Ceballos E., González S., Juarranz Á., Gilaberte Y. (2018). Clinical, histological and immunohistochemical markers of resistance to methyl aminolaevulinate photodynamic therapy in Bowen disease. Br. J. Dermatol..

[41] Cox N.H., Eedy D.J., Morton C.A. (2007). Therapy Guidelines and Audit Subcommittee; British Association of Dermatologists. Guidelines for management of Bowen’s disease: 2006 update. Br. J. Dermatol..

[42] De Haas E.R., Sterenborg H.J., Neumann H.A., Robinson D.J. (2007). Response of Bowen disease to ALA-PDT using a single and a 2-fold illumination scheme. Arch. Dermatol..

[43] Cervantes J.A., Zeitouni N.C. (2021). Photodynamic therapy utilizing 10% ALA nano-emulsion gel and red-light for the treatment of squamous cell carcinoma in-situ on the trunk and extremities: Pilot study and literature update. Photodiagn. Photodyn. Ther..

[44] Souza C.S., Felicio L.B., Ferreira J., Kurachi C., Bentley M., Tedesco A., Bagnato V. (2009). Long-term follow-up of topical 5-aminolaevulinic acid photodynamic therapy diode laser single session for non-melanoma skin cancer. Photodiagn. Photodyn. Ther..

[45] Kibbi N., Zhang Y., Leffell D.J., Christensen S.R. (2020). Photodynamic therapy for cutaneous squamous cell carcinoma in situ: Impact of anatomic location, tumor diameter, and incubation time on effectiveness. J. Am. Acad. Dermatol..

[46] Tarstedt M., Gillstedt M., Wennberg Larkö A.M., Paoli J. (2016). Aminolevulinic acid and methyl aminolevulinate equally effective in topical photodynamic therapy for non-melanoma skin cancers. J. Eur. Acad. Dermatol. Venereol..

[47] Alique-García S., Alique D., Company-Quiroga J., Sánchez A., Núñez A.H., Borbujo J. (2019). Treatment of Bowen’s disease with photodynamic therapy. Observational study in 171 patients with 5-aminolaevulinic acid (BF-200 ALA) and methyl aminolaevulinate (MAL). Photodiagn. Photodyn. Ther..

[48] Dragieva G., Hafner J., Dummer R., Schmid-Grendelmeier P., Roos M., Prinz B.M., Burg G., Binswanger U., Kempf W. (2004). Topical photodynamic therapy in the treatment of actinic keratoses and Bowen’s disease in transplant recipients. Transplantation.

[49] Perrett C.M., McGregor J.M., Warwick J., Karran P., Leigh I.M., Proby C.M., Harwood C.A. (2007). Treatment of post-transplant premalignant skin disease: A randomized intrapatient comparative study of 5-fluorouracil cream and topical photodynamic therapy. Br. J. Dermatol..

[50] Ratour-Bigot C., Chemidling M., Montlahuc C., Abirached G., Madjlessi N., Bullier C., Battistella M., Bagot M., Lebbe C., Basset-Seguin N. (2016). Squamous Cell Carcinoma Following Photodynamic Therapy for Cutaneous Bowen’s Disease in a Series of 105 Patients. Acta Derm. Venereol..

[51] Safar R., Alkhars A., Tallegas M., Korsaga-Some N., Machet L. (2019). Successful treatment for extensive Bowen’s disease using daylight-mediated photodynamic therapy. Acta Derm. Venereol..

[52] Martins C.C., Bakos R.M., Martins Costa M. (2020). Daylight photodynamic therapy for Bowen’s disease. An. Bras. Dermatol..

[53] Ahmady S., Nelemans P.J., Kelleners-Smeets N.W.J., Arits A.H.M.M., de Rooij M.J.M., Kessels J.P.H.M., Essers B.A.B., Mosterd K. (2024). Surgical excision versus topical 5% 5-fluorouracil and photodynamic therapy in treatment of Bowen’s disease: A multicenter randomized controlled trial. J. Am. Acad. Dermatol..

[54] Nakano A., Watanabe D., Akita Y., Kawamura T., Tamada Y., Matsumoto Y. (2011). Treatment efficiency of combining photodynamic therapy and ionizing radiation for Bowen’s disease. J. Eur. Acad. Dermatol. Venereol..

[55] Ko D.Y., Kim K.H., Song K.H. (2014). A randomized trial comparing methyl aminolaevulinate photodynamic therapy with and without Er:YAG ablative fractional laser treatment in Asian patients with lower extremity Bowen disease: Results from a 12-month follow-up. Br. J. Dermatol..

[56] Lu Y.G., Wang Y.Y., Yang Y.D., Zhang X.C., Gao Y., Yang Y., Zhang J.B., Li G.L. (2014). Efficacy of topical ALA-PDT combined with excision in the treatment of skin malignant tumor. Photodiagn. Photodyn. Ther..

[57] Cai H., Wang Y.X., Zheng J.C., Sun P., Yang Z.Y., Li Y.L., Liu X.Y., Li Q., Liu W. (2015). Photodynamic therapy in combination with CO_2_ laser for the treatment of Bowen’s disease. Lasers Med. Sci..

[58] Victoria-Martinez A.M., Martinez-Leborans L., Ortiz-Salvador J.M., Perez-Ferriols A. (2017). Treatment of Bowen Disease with Photodynamic Therapy and the Advantages of Sequential Topical Imiquimod. Actas Dermo-Sifiliogr..

[59] Genouw E., Verheire B., Ongenae K., De Schepper S., Creytens D., Verhaeghe E., Boone B. (2018). Laser-assisted photodynamic therapy for superficial basal cell carcinoma and Bowen’s disease: A randomized intrapatient comparison between a continuous and a fractional ablative CO2 laser mode. J. Eur. Acad. Dermatol. Venereol..

[60] Wu Y., Wang P., Zhang L., Wang B., Wang X. (2018). Enhancement of Photodynamic Therapy for Bowen’s Disease Using Plum-Blossom Needling to Augment Drug Delivery. Dermatol. Surg..

[61] Kim H.J., Song K.H. (2018). Ablative fractional laser-assisted photodynamic therapy provides superior long-term efficacy compared with standard methyl aminolevulinate photodynamic therapy for lower extremity Bowen disease. J. Am. Acad. Dermatol..

[62] Liu D., Wu L., Li J., Shi W., Li F., Su J., Huang K., Zhou Q., Zhao S., Chen M. (2019). Simple shaving combined with photodynamic therapy for refractory bowen disease. Photodiagn. Photodyn. Ther..

[63] Liu X., Wang J., Yu J., Xing W., Zhang J. (2023). Experience analysis of a combined photodynamic/electrodesiccation therapy in the treatment of 11 cases of large patches of Bowen’s disease. Photodiagn. Photodyn. Ther..

[64] Zhong S., Zhang R., Mei X., Wang L. (2020). Efficacy of photodynamic therapy for the treatment of Bowen’s disease: An updated systematic review and meta-analysis of randomized controlled trials. Photodiagn. Photodyn. Ther..

[65] Xue W.L., Ruan J.Q., Liu H.Y., He H.X. (2022). Efficacy of Photodynamic Therapy for the Treatment of Bowen’s Disease: A Meta-Analysis of Randomized Controlled Trials. Dermatology.

[66] Pérez-Pérez L., García-Gavín J., Gilaberte Y. (2014). Daylight-mediated photodynamic therapy in Spain: Advantages and disadvantages. Actas Dermosifiliogr..

[67] Fargnoli M.C., Kostaki D., Piccioni A., Micantonio T., Peris K. (2012). Dermoscopy in the diagnosis and management of non-melanoma skin cancers. Eur. J. Dermatol..

[68] Guida S., Alma A., Shaniko K., Chester J., Ciardo S., Proietti I., Giuffrida R., Zalaudek I., Manfredini M., Longo C. (2022). Non-Melanoma Skin Cancer Clearance after Medical Treatment Detected with Noninvasive Skin Imaging: A Systematic Review and Meta-Analysis. Cancers.

[69] Mun J.H., Park J.M., Song M., Jwa S.W., Kim H.S., Ko H.C., Kim B.S., Kim M.B. (2012). The use of dermatoscopy to monitor therapeutic response of Bowen disease: A dermatoscopic pathological study. Br. J. Dermatol..

[70] Mun J.H., Kim S.H., Jung D.S., Ko H.C., Kwon K.S., Kim M.B. (2010). Dermoscopic features of Bowen’s disease in Asians. J. Eur. Acad. Dermatol. Venereol..

[71] Teoh Y.L., Kuan L.Y., Chong W.S., Chia H.Y., Thng T.G.S., Chuah S.Y. (2019). The role of reflectance confocal microscopy in the diagnosis and management of squamous cell carcinoma in situ treated with photodynamic therapy. Int. J. Dermatol..

[72] Cinotti E., Perrot J.L., Labeille B., Douchet C., Mottet N., Cambazard F. (2014). Laser photodynamic treatment for in situ squamous cell carcinoma of the glans monitored by reflectance confocal microscopy. Australas. J. Dermatol..

[73] González-Guerra E., Taboada A.C., Muñoz L.C., Fructuoso A.I.S. (2023). Photodynamic therapy with BF-200 ALA gel for the treatment of actinic keratosis, Bowen’s disease and basal cell carcinoma in long term immunosuppressed patients after organ transplantation. Photodiagn. Photodyn. Ther..

[74] Ibbotson S.H., Wong T.H., Morton C.A., Collier N.J., Haylett A., McKenna K.E., Mallipeddi R., Moseley H., Rhodes L.E., Seukeran D.C. (2019). Adverse effects of topical photodynamic therapy: A consensus review and approach to management. Br. J. Dermatol..

